# Comparative Analysis of Force-Sensitive Resistors and Triaxial Accelerometers for Sitting Posture Classification

**DOI:** 10.3390/s24237705

**Published:** 2024-12-02

**Authors:** Zhuofu Liu, Zihao Shu, Vincenzo Cascioli, Peter W. McCarthy

**Affiliations:** 1 The Higher Educational Key Laboratory for Measuring and Control Technology and Instrumentations of Heilongjiang Province, Harbin University of Science and Technology, Harbin 150080, China; 2220600021@stu.hrbust.edu.cn; 2 Murdoch University Chiropractic Clinic, Murdoch University, Murdoch 6150, Australia; v.cascioli@murdoch.edu.au; 3 Faculty of Life Science and Education, University of South Wales, Treforest, Pontypridd CF37 1DL, UK; peter.mccarthy@southwales.ac.uk; 4 Faculty of Health Sciences, Durban University of Technology, Durban 1334, South Africa

**Keywords:** sitting posture, force-sensitive resistor, triaxial accelerometers, classification algorithm, sensor verification, accuracy, computational efficiency

## Abstract

Sedentary behaviors, including poor postures, are significantly detrimental to health, particularly for individuals losing motion ability. This study presents a posture detection system utilizing four force-sensitive resistors (FSRs) and two triaxial accelerometers selected after rigorous assessment for consistency and linearity. We compared various machine learning algorithms based on classification accuracy and computational efficiency. The k-nearest neighbor (KNN) algorithm demonstrated superior performance over Decision Tree, Discriminant Analysis, Naive Bayes, and Support Vector Machine (SVM). Further analysis of KNN hyperparameters revealed that the city block metric with K = 3 yielded optimal classification results. Triaxial accelerometers exhibited higher accuracy in both training (99.4%) and testing (99.0%) phases compared to FSRs (96.6% and 95.4%, respectively), with slightly reduced processing times (0.83 s vs. 0.85 s for training; 0.51 s vs. 0.54 s for testing). These findings suggest that, apart from being cost-effective and compact, triaxial accelerometers are more effective than FSRs for posture detection.

## 1. Introduction

In recent years, a sedentary lifestyle has become the norm for many, bringing with it a host of health concerns. Spending excessive hours—more than six—in a sedentary state, particularly with poor postures, can result in physical harm and a myriad of health issues [1,2,3]. Research shows that back pain caused by prolonged sitting is widespread among the population aged 25 to 45, impacting both genders and consequently affecting individuals during their most productive work years [4,5,6]. Sedentary-related spinal diseases are a major contributor to sick leave and early retirement, causing significant economic impact [7,8,9]. Globally, nearly two million people annually develop musculoskeletal disorders from prolonged improper sitting, including conditions like cervical spondylosis [10] and intervertebral disc damage [11,12]. Early detection and preventative measures are crucial in reducing the risk of musculoskeletal disorders and skin ulcer formation in individuals who are sedentary for long periods due to limited mobility. Monitoring changes in posture [13,14,15] and seating ergonomics, along with tailored interventions or advice [16,17], can play a vital role in addressing this global health challenge.

In recent years, there has been a surge in interest in monitoring sitting posture through either objective or subjective means. While specific questionnaires have often been utilized, they have inherent limitations, such as being subjective and incapable of providing continuous monitoring of sitting postures [16]. Consequently, there is a growing focus on the development of postural detection devices that can objectively measure and quantify the posture of seated individuals.

These devices can be categorized into three main groups based on the level of intrusion of the measurement technique: wearable sensors, camera or video sensors, and pressure sensors [8,10,16]. A comparison of the various sensors applied to sitting posture detection is presented in Table 1 to help in understanding the relative pros and cons of each method.

Compared with camera sensors, both wearable and pressure sensors pose a lower risk of privacy disclosure and demonstrate greater resilience to environmental interference [26]. Additionally, there have been both commercial and custom-tailored solutions available. For instance, ActiGraph (ActiGraph L.L.C., Pensacola, FL, USA) and activPAL (PAL Technologies Ltd., Glasgow, Scotland, UK) are well-established options for wearable sensors, while the XSensor (XSensor Technology Co., Calgary, AB, Canada) mesh pad is a recognized choice for pressure measurement [8,13]. With the advancement in MEMS (Micro-Electro-Mechanical System) techniques, triaxial accelerometers and force-sensitive resistors (FSRs) have become quintessential sensors for motion detentions [8,23]. However, a comprehensive performance comparison between these two kinds of sensors specifically for sitting posture detection remains sparse in the literature.

In this study, we aim to propose an economical and efficient sitting posture detection system by comparing the performance of accelerometers against FSRs. Various sitting postures are classified using classification models that leverage data collected from both sensors. The structure of the paper is organized as follows: Section 2 details sensor verification procedures, the system configuration, and the experimental setup. Section 3 presents the experimental results. Section 4 offers an analysis and discussion of these results. Finally, Section 5 summarizes our key findings.

## 2. Materials and Methods

Although sensors available in the market are subjected to batch sampling by their manufacturers, it is essential to evaluate their specifications, such as consistency and linearity, especially when multiple sensors are used in conjunction for data collection. For the subsequent experimental tests, an Arduino UNO (Taobao Co., Hangzhou, China) was utilized as the data acquisition and transmission unit, operating at a sampling frequency of 10 Hz.

### 2.1. FSR Verification

**Figure 1:**
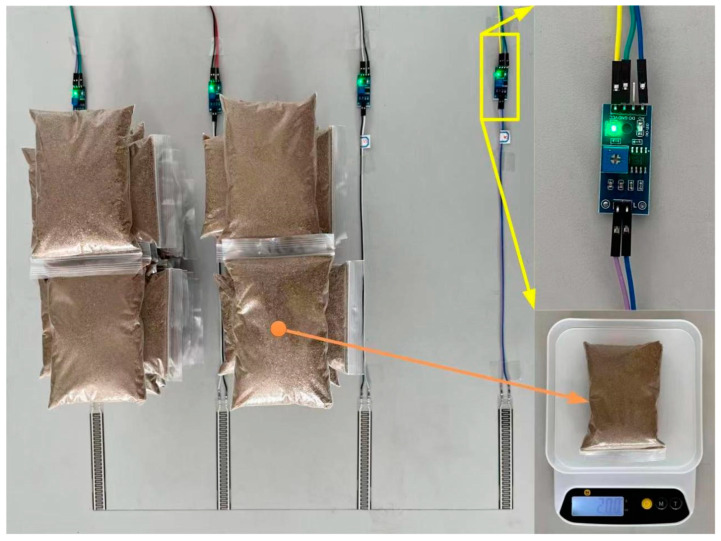
FSR performance verification using sandbags to simulate the loading force.

Four FSRs (FSR408, Interlink Electronics, Camarillo, CA, USA) were securely affixed to the flat surface of an experimental table using transparent adhesive tape. To simulate the loading force, a series of 200 g sandbags were incrementally placed on the sensors, as shown in Figure 1. The output pins from these four FSRs were connected to the Analog-to-Digital Converter (ADC) interface of the Arduino UNO, employing standard voltage dividers to maintain compatibility with the ADC’s input requirements.

Following an initial period (2 s) for output stabilization, the raw sensor data were transmitted to a laptop for further analysis. This data transmission was facilitated by a Universal Serial Bus (USB) cable, which connected the Arduino UNO to the laptop. To mitigate the effects of noise interference, a data smoothing filter was employed, and the loading forces ranged from 200 g to 3000 g.

### 2.2. Accelerometer Verification

**Figure 2:**
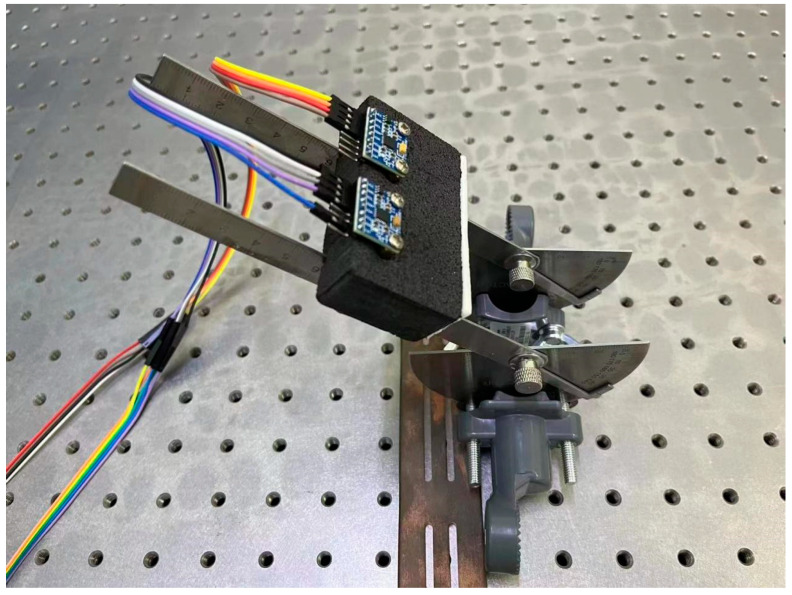
Accelerometer evaluation test using a custom-made rotating device placed on the optical platform.

To validate the triaxial accelerometer (ADXL345, Analogy Devices Inc., Wilmington, MA, USA), we utilized a custom-designed rotating device (Figure 2) capable of rotating through a 180° arc. The device was mounted on the optical platform to enhance the stability of the testing system. Each axis of the two accelerometers was thoroughly positioned along the rotating device, starting with the *x*-axis, then proceeding to the *y*-axis, and finally, the *z*-axis. For each axis alignment, the rotating device with the accelerometers was turned in increments of 5°.

The digital output pins of the two accelerometers were connected to an Arduino UNO using the I2C (Inter-Integrated Circuit) communication protocol, which facilitates streamlined data transmission over just two wires: one for clock signals and the other for data. To differentiate between the two accelerometers, their I2C addresses were set to 0 × 69 and 0 × 68. This was achieved by connecting the AD0 pin of one accelerometer to the +3.3 V output pin of the Arduino UNO, assigning it the address 0 × 69, while the second accelerometer’s AD0 pin was left unconnected, defaulting its address to 0 × 68. This configuration allows for individual addressing of each accelerometer, ensuring accurate data interpretation throughout the collection process. Similar to the FSR evaluation trials, the accelerometers’ data were transmitted to a laptop using a USB cable.

### 2.3. Sitting Posture Detection System

**Figure 3:**
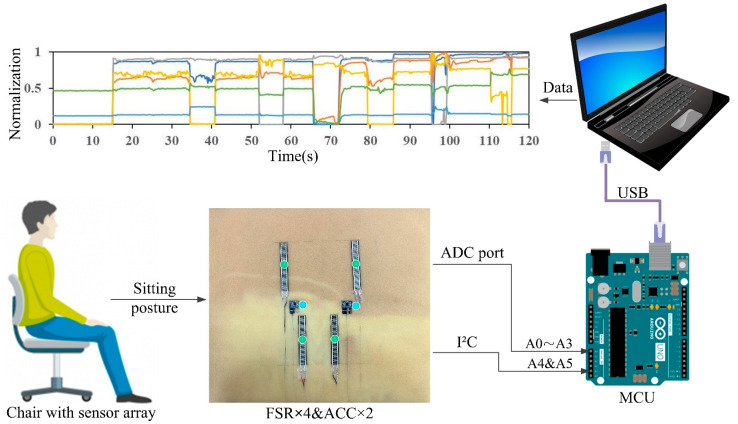
Configuration of the sitting posture detection system consisting of four FSRs and two triaxial accelerometers (sensors have been labeled numerically and ACC refers to accelerometer).

Four FSRs were mounted on a foam cushion using self-adhesive tape, targeting the areas beneath the left and right thighs and the left and right ischial tuberosities. These specific regions were selected due to their prominence upon contact with the cushion surface, which is crucial for accurate pressure measurement. Furthermore, two accelerometers were positioned at the cushion’s center to capture movement data. The sensor placement was instructed by the methodologies of previous studies [8,13,16]. The FSR sensors were interfaced with the analog ports, while the accelerometers were connected to the digital ports (Figure 3). The collected data were sampled and transmitted through a USB cable to a laptop, where they were subsequently stored in a spreadsheet for more comprehensive analysis.

### 2.4. Participant

Participants for the study were carefully selected to ensure they were free from pain and in good health. The exclusion criteria were designed to reduce the potential for confounding factors that could skew the results, which included the following [13]:

(1) Any indication of back pain or sitting-related disorders;
(2) Consumption of alcohol prior to the experiment;
(3) Any muscle or bone injuries within the last four months;
(4) A history of deep vein thrombosis;
(5) Any surgical procedures within the past year;
(6) Blood clotting disorders;
(7) Current use of medications such as aspirin, warfarin, or heparin.

A total of thirty university students (15 males and 15 females) participated in the sitting trials. The basic demographic information of the participants can be found in Table 2. The trials took place from 12 May to 20 May 2024. The study was approved by the Faculty Research Committee (No. 202301) and was conducted in accordance with the guidelines outlined in the Declaration of Helsinki.

### 2.5. Statistical Analysis

The data were analyzed using Matlab 2022a (MathWorks, Natick, MA, USA) and Excel 2016 (Microsoft, Seattle, WA, USA). This process encompassed the computation of the Spearman correlation coefficient to evaluate the relationship between FSRs and accelerometers. Additionally, a one-way ANOVA or *t*-test was employed to assess the consistency within the datasets gathered from the FSRs and accelerometers, respectively. Linear regression analysis was also conducted specifically for the FSRs. A significance level of <0.05 was considered indicative of statistical significance.

### 2.6. Sitting Postures Methodology

#### 2.6.1. Static Postures Analysis

In a comparative study, all 30 participants using the sitting posture detection system were instructed to adopt seven static sitting positions that reflect typical daily habits. These positions included sitting upright, leaning left, leaning right, leaning forward, leaning backward, crossing the left leg, and crossing the right leg. Each posture was held for 20 s before transitioning to the next one. The raw data captured by the FSRs and accelerometers were normalized before further processing.

#### 2.6.2. Dynamic Postures Analysis

In addition to the seven static sitting postures, the study also examined the characteristics of two dynamic sitting positions: fidgeting with the left leg and fidgeting with the right leg. These dynamic postures provide insights into the correlation between the FSRs embedded in the cushion surface and the triaxial accelerometers placed in the cushion slot. Similar to the static trials, each dynamic posture was held for 20 s, and Spearman correlation analysis was employed to evaluate the relationship between the FSRs and accelerometers.

## 3. Results

### 3.1. Performance of FSR

**Figure 4:**
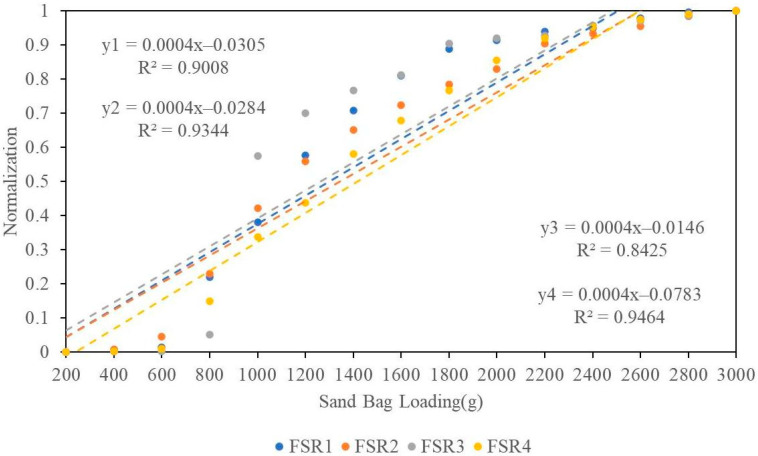
Normalized data from four FSR sensors (abbreviated by FSR1 to FSR4 in accordance with the numerical labels in Figure 3) in response to the loading force simulated by sandbags, along with linear fitting functions (from y1 to y4) and corresponding correlation coefficients.

The one-way ANOVA conducted on the four FSRs did not yield any significant differences among them (*p* > 0.05). Additionally, a linear curve fitting analysis (Figure 4) was performed on the sampled data, revealing a strong positive linear relationship (R2 > 0.8) between the input and output.

### 3.2. Performance of Accelerometer

**Figure 5:**
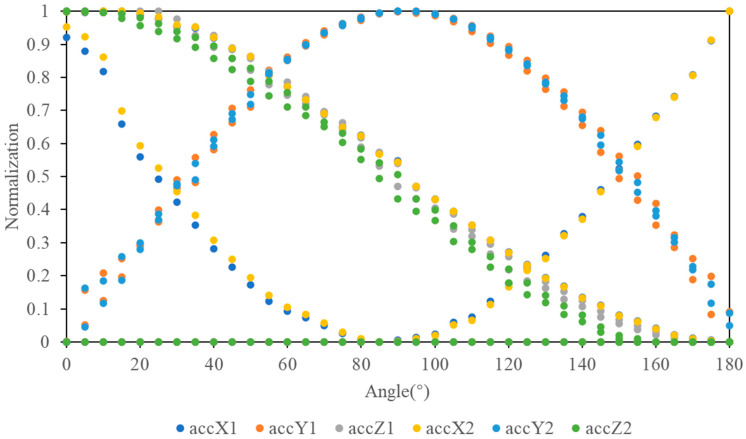
Normalized accelerometer data collected during rotation from 0 to 180° around each of the three axes in 5° intervals. According to the numerical labels in Figure 3, accX1 indicates the *x*-axis output of the first accelerometer, accX2 is the *x*-axis output of the second accelerometer, accY1 and accY2 correspond to *y*-axis outputs of the two accelerometers, and accZ1 and accZ2 are the *z*-axis outputs.

A *t*-test was conducted on each axis of the two accelerometers, indicating no significant difference (*p* > 0.05). Moreover, the curves (Figure 5) demonstrated strong consistency as the sensors were rotated from 0° to 180°.

### 3.3. Responses of FSRs and Accelerometers to Different Sitting Postures

**Figure 6:**
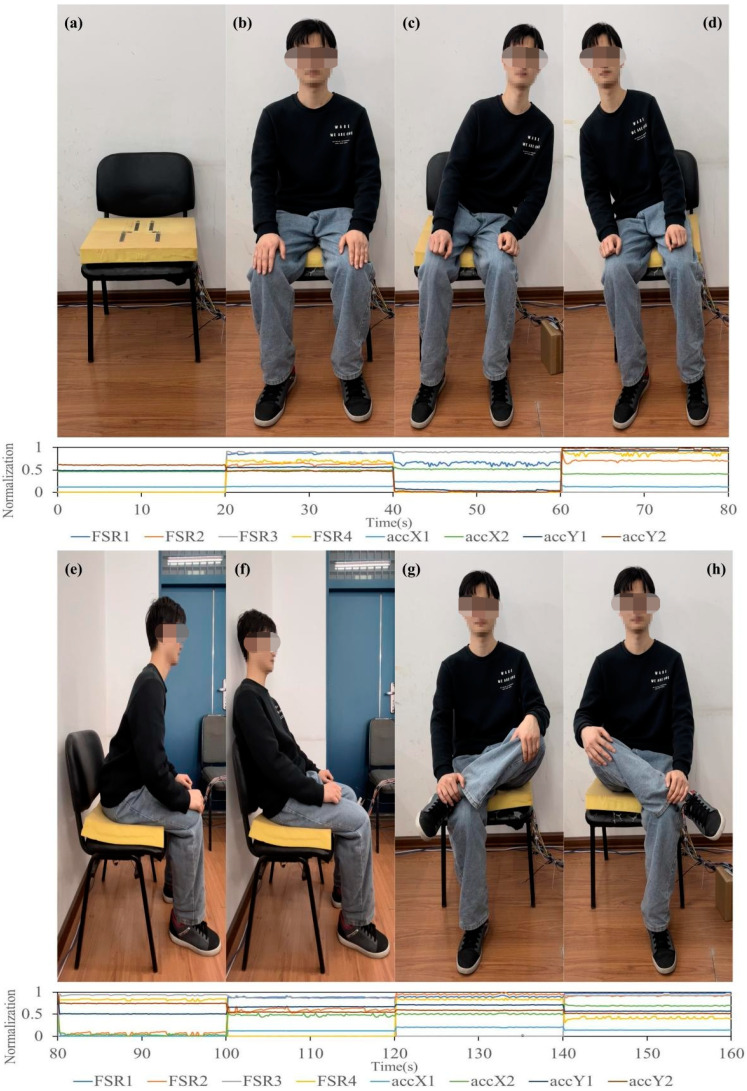
Variations in the normalized readings of four FSRs and two accelerometers as a randomly selected participant transitions through a series of sitting postures: (**a**) vacant (**b**) sitting upright, (**c**) leaning left, (**d**) leaning right, (**e**) leaning forward, (**f**) leaning backward, (**g**) crossing the left leg, and (**h**) crossing the right leg.

After normalizing the data in the time domain, the response curves from a randomly selected participant—recorded by four FSRs and two accelerometers—are presented in Figure 6.

**Figure 7:**
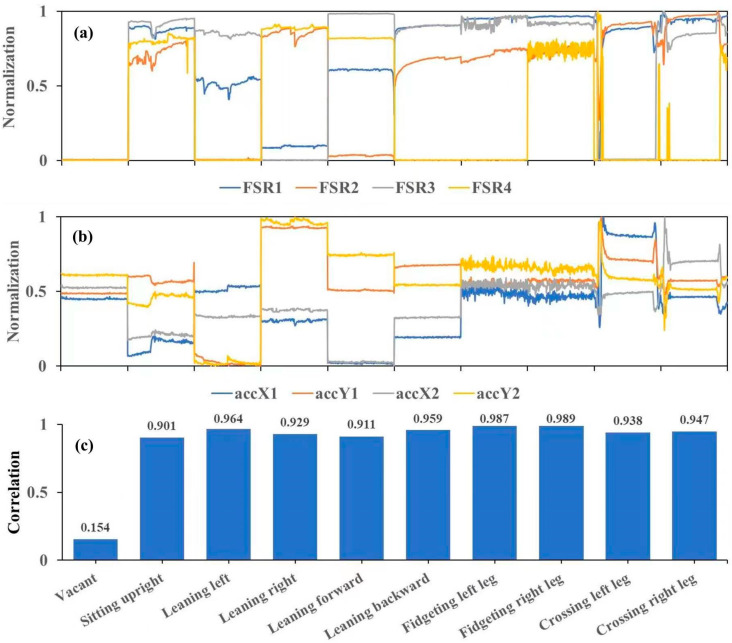
Illustration of the normalized curves of FSRs and accelerometers corresponding to different sitting postures and the maximum correlation coefficients between them. (**a**) Normalized curves of the FSRs. (**b**) Normalized curves of the accelerometers. (**c**) The maximum correlation coefficients between FSRs and accelerometers. FSR1 to FSR4 represent the four FSRs, while accX1 indicates the *x*-axis output of the first accelerometer, accX2 is the *x*-axis output of the second accelerometer, and accY1 and accY2 correspond to the *y*-axis outputs of the two accelerometers.

Figure 7 illustrates the correlation coefficients between the FSRs and accelerometers for both an empty cushion (vacant) and nine different sitting postures. The correlation coefficients between the FSRs and the accelerometers show there are strongest correlations (>0.8) between them in every distinct sitting posture.

### 3.4. Sitting Posture Classification

To classify different sitting postures, several popular classification algorithms were employed, including Decision Tree, Discriminant Analysis, Naive Bayes, SVM (support vector machine), and KNN (k-nearest neighbor) algorithms. To validate the accuracy of different models, sampling data were divided into training (70%) and testing (30%) groups.

For the force-sensitive resistors, we choose FSR1, FSR2, FSR3, and FSR4 as input features to the classifiers. As for the accelerometers, according to the analysis in Section 3.3., we choose accX1, accY1, accX2, and accY2 as features as they effectively represent the dynamic characteristics of the sitting postures while ignoring accZ1 and accZ2 as they have little response to sitting posture changes. We excluded accZ1 and accZ2 due to their minimal response to changes in sitting posture. Table 3 highlights all results with classification accuracy exceeding 90% for both training and testing data. Upon reviewing the performance of these classification models, it is evident that the KNN algorithm stands out, demonstrating excellent performance in terms of both accuracy and running time during the training and testing phases.

## 4. Discussion

In this paper, we have developed a posture detection system that not only compares two types of sensors (FSRs and triaxial accelerometers) but also integrates them within a broader machine learning framework. This system-level approach has practical applications in the field of healthcare. Our findings on the superior performance of triaxial accelerometers over FSRs set a new benchmark for sensor selection in similar applications. Additionally, we provide detailed insights into the hyperparameter tuning of the k-nearest neighbor (KNN) algorithm, which is a significant contribution to the field as it optimizes the posture detection process.

Although previous studies [27,28,29,30] have utilized either FSR arrays or accelerometers to examine sitting postures, there is a dearth of literature exploring the combined application of FSRs and accelerometers. A notable exception [27] employed sixteen FSRs and one IMU (Initial Motion Unit) to classify seven postures with an average accuracy of 90.9%. In contrast, our study expands the scope by comparing nine postures—seven static ones as described in [27] and two dynamic ones (left/right leg fidgeting)—and achieved remarkable accuracy rates of 99.4% for training and 99.0% for testing, facilitated by the optimal KNN classifier and the use of two accelerometers. This underscores the significance of sensor placement and feature extraction techniques.

### 4.1. Comparison Between FSR and Accelerometer

By analyzing the normalized time-domain curves of FSRs and triaxial accelerometers, along with their correlation coefficients (Figure 7), we can preliminarily conclude that there is a consistent correlation between the data from FSRs and accelerometers across different sitting postures. This indicates that changes in a subject’s sitting posture correspond to changes in both data sets. This consistency suggests that triaxial accelerometers could effectively replace FSRs for assessing sitting posture, offering a new approach for future studies. Additionally, accelerometers have a broader measurement range and better linearity compared to FSRs, which only exhibit approximate linearity within a narrow range. Furthermore, it might be possible that the larger person might push the FSR outside the linear range.

### 4.2. Classification Algorithms

**Figure 8:**
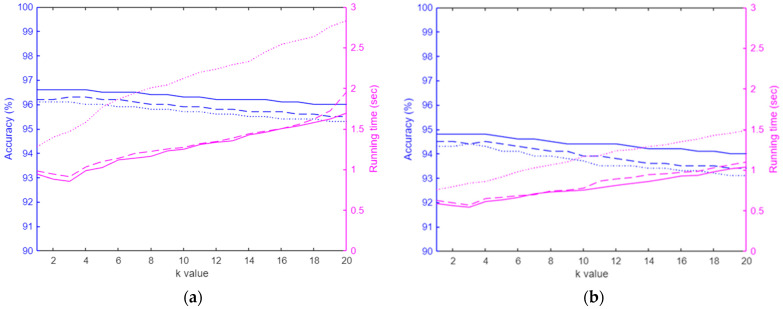

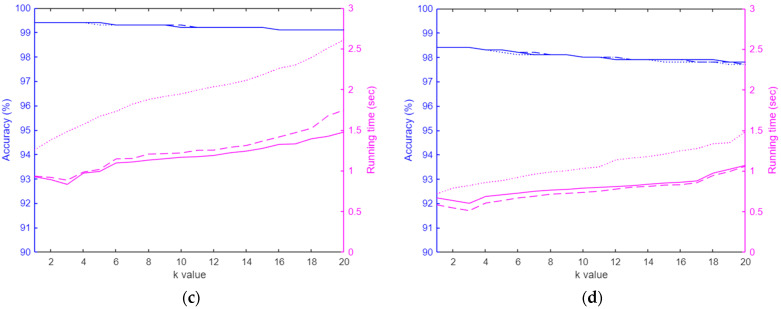
Comparison of various hyperparameters (k-values and metric functions) for K-Nearest Neighbors (KNN). Classification results for FSRs: (**a**) training data results displayed and (**b**) testing data results. Classification results for the accelerometers, (**c**) training data results, and (**d**) testing data results. The ‘solid line’ represents results using the City Block metric, the ‘dashed line’ corresponds to the Euclidean metric, and the ‘dotted line’ indicates the Cubic metric. Blue is used to depict accuracy comparisons, while magenta represents running time.

As KNN outperformed other classifications in terms of accuracy and computational cost (Table 3), we further investigated the influence of its two hyperparameters (metrics and k-value) on classification performance. The results (Figure 8) indicate that the city block metric function achieved the highest accuracy for both FSR training and testing data, at 96.6% and 95.4%, respectively. For computational efficiency, K = 3 was selected, yielding the fastest running times of 0.85 s for training and 0.54 s for testing. For the accelerometers, the three metric functions achieved the same and highest accuracy of 99.4% for training data and 99.0% for the testing data when K changed from 1 to 3. Notably, the city block metric again provided the fastest running times of 0.83 s for training and 0.51 s for testing when K = 3.

**Figure 9:**
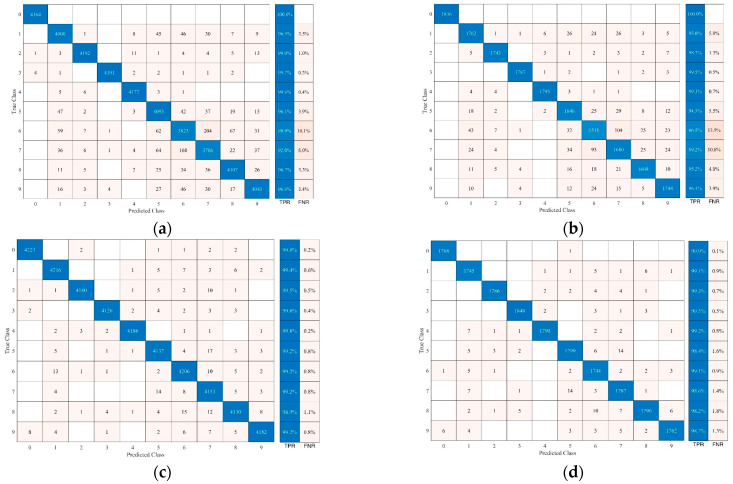
Confusion matrix in which 0—vacant, 1—sitting upright, 2—leaning left, 3—leaning right, 4—leaning forward, 5—leaning backward, 6—fidgeting left leg, 7—fidgeting right leg, 8—crossing left leg, 9—crossing right leg. (**a**) FSR training data, (**b**) FSR testing data, (**c**) accelerometer training data, and (**d**) accelerometer testing data. For all the comparisons, hyperparameters of KNN are city block metric function and K = 3.

Additionally, the confusion matrix for KNN across nine sitting postures was calculated (Figure 9) with K = 3 and city block selected as the metrics. It reveals that FSR struggles to distinguish fidgeting, achieving lower performance in both training (89.9% for fidgeting left leg and 92% for fidgeting right leg) and testing (86.5% for fidgeting left leg and 89.2% for fidgeting right leg) phases, while accelerometers demonstrate better performance in this regard (training: 99.2% for fidgeting both left and right leg, testing: 99.1% and 98.6% for fidgeting left and right leg, respectively).

The comparisons indicate that utilizing a smaller number of sensors, specifically two triaxial accelerometers, yields better classification accuracy than using four FSR arrays, thereby enhancing the robustness of the measurement system. Additionally, triaxial accelerometers are significantly more cost-effective, priced at approximately USD 1 each compared to around USD 5 for FSRs, which helps to reduce overall system costs. Furthermore, the digital interface of the accelerometers enhances data transmission stability and simplifies peripheral circuit design in comparison to FSRs.

### 4.3. Limitation

This pilot study focused on healthy young individuals and short sitting durations, underscoring the need for future research to include a more diverse population in terms of age, body mass, and height to evaluate model performance over longer sitting times. Considering individuals with higher BMI is particularly important for assessing clinical effectiveness [8,31]. While the current classification results are promising with a limited number of participants, exploring optimal accelerometer placements remains essential [23,32]. In addition, we plan to expand our future work to include dynamic activities (e.g., typing, using a mobile device, or leaning forward to reach for objects) to evaluate the system’s performance in a more realistic and diverse series of scenarios. Although various machine learning algorithms were compared, developing more explainable classification methods that incorporate sensor optimization is crucial [8,33,34]. Additionally, accelerometer-based sitting posture detection faces limitations in measuring small deformations in rigid cushions, such as wooden chairs. However, techniques from the construction industry, like linear transformation matrices, could provide valuable insights [35]. Moreover, many office chairs and public transport seats are made from foam-like materials, making them suitable for embedding accelerometers to detect sitting postures.

## 5. Conclusions

This paper investigates the use of FSRs and triaxial accelerometers for sitting posture classification, highlighting the importance of accurate data analysis and model calibration for both sensor types. Experiments comparing two accelerometers with four FSRs revealed that the accelerometers not only match but often outperform the FSRs in recognizing diverse sitting postures. Analysis of classification results demonstrates that accelerometers consistently achieve higher accuracy rates and lower computational costs. These findings suggest a more cost-effective sitting posture detection system and provide innovative insights for advancing sitting posture classification technology.

## Figures and Tables

**Table 1:** Typical objective measures used for sitting posture detection.

Sensor Type	Advantage	Disadvantage
Wearable sensors * [18,19,20]	Thin and tiny in size Good portability	Intrusiveness can lead to discomfort for individuals during wear Challenges in cleaning can arise with the fabrication of garments Regular recalibration may be necessary to account for potential calibration drift Difficulty in maintaining consistent contact location
Camera sensors [17,21,22]	RGB + depth sensor provides 3D images	Vulnerable to environmental factors such as lighting and image occlusion Risk of privacy invasion
Pressure sensors [8,13,23]	Facilitate the construction of a sensing array with ease Eliminate the need for bulky electronics in the implementation process A certain degree of portability	Requires traceable calibration before use to ensure accuracy A grid-based pressure mat is costly and entails high computational expenses There is a risk of electronic wire damage, particularly at the connection points, due to regular bending/distortion

* Wearable sensors include three parts [18,24,25]: (1) Inertial Measurement Units (IMUs) are pivotal in posture detection due to their ability to measure linear acceleration and angular velocity relative to the human body; (2) EcoFlex, a material known for its hyperelastic properties, are capable of translating subtle deformations into resistance variations; (3) Conductive Fabric, which is indicative of an individual’s sitting posture, can be integrated into seating surfaces or wearable devices to measure pressure distribution.

**Table 2:** Demographic information of all the subjects reported as mean ± standard deviation.

Participants Number	Sex	Age (Year)	Height (cm)	Weight (kg)
15	Male	24.40 ± 2.59	174.27 ± 6.65	73.53 ± 10.90
15	Female	23.20 ± 2.31	166.17 ± 6.56	56.53 ± 9.16
30	Male/Female	23.80 ± 2.48	170.22 ± 7.69	65.03 ± 13.14

**Table 3:** Accuracy and computational cost of different classification algorithms using the preset hyperparameters.

Data Category	Classification Algorithms	Parameters	Training Set		Testing Set	
			Accuracy (%)	Training Time (s)	Accuracy (%)	Testing Time (s)
FSRs	Decision Tree	Max splits: 100 Split criterion: Gini index	71.8	2.4124	70.2	4.299
		Max splits: 20 Split criterion: Gini index	58.5	1.2109	56.7	0.9742
		Max splits: 4 Split criterion: Gini index	42.6	1.1983	41.8	0.7447
	Discriminant Analysis	DiscrimType: Linear Covariance: Constant	50.8	0.78712	49.2	1.2891
		DiscrimType: Quadratic Covariance: Unconstrained	53.7	2.1054	53.2	0.9063
	Naive Bayes	DistributionNames: Gaussian	50.8	1.3587	50.8	1.3541
		DistributionNames: Kernel KernelType: Epanechnikov Support: Unbounded	63.7	67.957	63.0	19.129
	SVM	Linear kernel function	55.9	2899.6	56.9	257.94
		Quadratic kernel function	65.6	9163.1	66.9	2602.8
		Cubic kernel function	60.5	9465.3	68.4	2889.6
		Gaussian Kernel function Kernel scale: 2	76.6	106.41	73.9	20.834
	KNN	**Metrics: Euclidean; k = 10**	**94.5**	**1.2803**	**92.0**	**0.7867**
		**Metrics: City block; k = 10**	**96.3**	**1.5597**	**95.2**	**0.7638**
		**Metrics: Cubic; k = 10**	**94.2**	**2.3192**	**91.5**	**1.2753**
ACCs	Decision Tree	Max splits: 100 Split criterion: Gini index	67.7	2.1993	68.2	1.1988
		Max splits: 20 Split criterion: Gini index	47.1	1.2089	45.3	1.0929
		Max splits: 4 Split criterion: Gini index	34.9	1.1564	35.1	1.2004
	Discriminant Analysis	DiscrimType: Linear Covariance: Constant	46.9	1.321	47.0	0.9289
		DiscrimType: Quadratic Covariance: Unconstrained	50.7	1.2721	51.5	0.9724
	Naive Bayes	DistributionNames: Gaussian	47.0	1.8406	47.5	1.1575
		DistributionNames: Kernel KernelType: Epanechnikov Support: Unbounded	63.8	69.848	57.3	19.898
	SVM	Linear kernel function	48.8	2146.1	50.0	250.39
		Quadratic kernel function	63.1	12,809	63.8	2828.3
		Cubic kernel function	71.5	11,728	85.8	2963.3
		Gaussian Kernel function Kernel scale: 2	86.5	161.69	80.5	23.544
	KNN	**Metrics: Euclidean; k = 10**	**98.9**	**1.6779**	**98.2**	**1.4447**
		**Metrics: City block; k = 10**	**98.9**	**1.0532**	**98.2**	**0.6503**
		**Metrics: Cubic; k = 10**	**98.9**	**2.1747**	**98.2**	**1.1119**

## Data Availability

The data presented in this study are available on request from the corresponding author.
